# A novel and simple scoring system for assessing the indication for catheter ablation in patients with atrial fibrillation: The HEAL‐AF Score

**DOI:** 10.1002/joa3.12429

**Published:** 2020-09-02

**Authors:** Takayuki Otsuka, Shinya Suzuki, Takuto Arita, Naoharu Yagi, Takanori Ikeda, Takeshi Yamashita

**Affiliations:** ^1^ Department of Cardiovascular Medicine The Cardiovascular Institute Tokyo Japan; ^2^ Department of Cardiovascular Medicine Toho University Graduate School of Medicine Tokyo Japan

**Keywords:** asymptomatic atrial fibrillation, atrial fibrillation, catheter ablation, elderly, heart failure, scoring system

## Abstract

**Introduction:**

A scoring system to determine indications for catheter ablation (CA) in atrial fibrillation (AF) is desired.

**Methods and Results:**

Among 2898 consecutive patients with AF, CA was performed in 938 (32.4%). A new HEAL‐AF score has been developed by six variables, all of which were independently associated with CA by multivariate analysis and for each 1 point was assigned: heart failure ≥ NYHA II, elderly patients (age ≥75 years), asymptomatic AF, long‐standing persistent AF, atrial dilation (left atrial diameter ≥ 50 mm), and female sex. Low HEAL‐AF score was associated with high incidence of CA performance (52.0% for 0, 36.5% for 1, 15.1% for 2, and 5.6% for ≥ 3) and the predictive capability of this score by AUC of ROC curve was 0.720 (95% CI 0.701‐0.739, *P *< .001). The rates of freedom from AF/AT recurrence were 73.2% in HEAL‐AF score 0, 71.0% in 1, 60.0% in 2, and 50.0% in ≥ 3 (log‐rank test, *P* = .004). HEAL‐AF score 2 and ≥ 3 were significantly associated with recurrence of atrial tachyarrhythmia as compared with HEAL‐AF 0 (HR 1.755, *P* = .002, and HR 2.211, *P* = .007, respectively).

**Conclusions:**

A new HEAL‐AF score was associated with patient indication for and the recurrence of atrial tachyarrhythmia after CA in patients with AF. Prescription of CA should be considered carefully in AF patients with HEAL‐AF score of 2 and ≥ 3.

## Graphical abstract

The HEAL‐AF score consists of six simple clinical variables (1 point for each of heart failure ≥ NYHA II, age ≥ 75 years, asymptomatic AF, and long‐standing persistent AF, left atrial diameter ≥ 50 mm, and female sex). The HEAL‐AF score was significantly associated with not only patient selection for catheter ablation (CA) but also complex ablation strategy and recurrence of AF after CA.

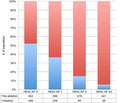

## 1 INTRODUCTION

Catheter ablation (CA) of atrial fibrillation (AF) has been shown to be a more effective rhythm control strategy for AF patients than antiarrhythmic drug (AAD) therapy in several clinical trials.^1^, ^2^, ^3^ However, these trials enrolled mostly young and robust patients with symptomatic paroxysmal AF, and the safety and efficacy of CA have not been well established for other types of AF patients. For individualized assessment to select patients for CA, there are a number of clinical characteristics to be considered, such as type and duration of AF, degree of symptoms, presence or absence of significant heart failure (HF), age, left atrial (LA) size, number of failed AADs, likelihood of complications, and patient preference. Despite recent revisions of the guidelines,^4^, ^5^, ^6^, ^7^ there remains a debate how to select appropriate patients for CA, thereby confusing general cardiologists and primary care physicians.

Several scoring systems, such as CHADS2 score^8^ and CHA2DS2‐VASc score,^9^ have played a significant role for selecting the AF patients who needs anticoagulation therapy for prevention of thromboembolic events. Similarly, a scoring system to determine the indication for CA in AF, if any, would be useful, but currently has not been well established. The aim of the present study was to develop a scoring system for the patient selection for CA in patients with AF using data from a single hospital‐based cohort, and to evaluate whether the score is associated with the rhythm outcome after CA.

## 2 METHODS

### 2.1 Subjects

The Shinken Database was established for all new patients who visited The Cardiovascular Institute, Tokyo, Japan (Shinken is an abbreviation of the name of the hospital in Japanese). Patients with active cancer and foreign travelers were excluded. The principal aim of this hospital‐based database is to survey the prevalence and prognosis of cardiovascular diseases in a cardiovascular‐specialized hospital, including arrhythmic specialists, in an urban area of Japan.^10^, ^11^ The registry was started in June 2004, and thereafter, patients have been continually registered in the database. The data in the present study were derived from this database between April 2009 and March 2017, when the annual number of AF patients who underwent CA exceeded 50 cases per year. During this period, 12 760 patients were newly registered in the total database, among whom AF was diagnosed in 2898.

In the present study, AF was diagnosed by electrocardiography, including 12‐lead surface electrocardiograms (ECG) and 24‐hour Holter ECG performed within 3 months after the initial visit, and by a medical history of AF from the referring physician, and new‐onset AF that occurred> 3 months after the initial visit was not included in the diagnosis of AF. Any AF episodes within 3 months after the initial visit, including first detected AF and post‐operative AF, were diagnosed as AF. Definitions of paroxysmal AF, persistent AF, and long‐standing persistent AF were according to the ACC/AHA/ESC guidelines^4^: paroxysmal AF was defined as AF that terminates spontaneously or with intervention within 7 days of onset; persistent AF was defined as continuous AF that lasted more than 7 days; and long‐standing persistent AF was defined as continuous AF with a duration > 12 months. The definition of asymptomatic AF was the lack of the usual AF‐related symptoms, including palpitations, shortness of breath, chest oppression, dizziness, etc The concomitance of acute or chronic HF in patients with asymptomatic long‐standing persistent AF was also categorized as asymptomatic AF.

### 2.2 Catheter ablation

Our ablation protocol was described previously.^12^ All patients had anticoagulation therapy for more than 3 weeks before ablation and underwent transesophageal echocardiography to exclude atrial thrombus within 3 days before ablation. All AADs were discontinued for at least 5 half‐lives before the procedure, except amiodarone. All procedures were performed under deep sedation using fentanyl and continuous infusion of propofol. Briefly, pulmonary vein (PV) isolation (PVI) was performed by circumferential applications of radiofrequency (RF) energy at each PV antrum with a 3‐dimensional mapping system (CARTO XP/CARTO 3; Biosense Webster, or Ensite NavX; St. Jude Medical). An open‐irrigated ablation catheter was advanced into the left atrium (LA) via a conventional short sheath between April 2009 and October 2014. An open‐irrigated ablation catheter with or without contact force sensor was advanced into the LA via a steerable sheath (Agilis; St. Jude Medical) after November 2014. Linear ablation for isolation of the LA posterior wall was also added in all patients with non‐paroxysmal AF after November 2014. RF energy was delivered at a maximum power of 40W, with a target temperature of 43°C. After completion of PVI and linear ablation at the cavotricuspid isthmus, the inducibility of atrial tachyarrhythmia was assessed sequentially with electrophysiological and pharmacological methods as described previously.^12^ If AF or atrial tachycardia (AT) lasting more than 5 minutes was induced by atrial pacing, additional ablation including targeting complex fractionated atrial electrograms (CFAE) and LA linear ablation were performed. Repetitive non‐PV ectopic beats were also eliminated if these were induced with isoproterenol infusion. Finally, the dormant conduction provoked by administration of adenosine triphosphate (ATP, 20 mg) was ablated at the end of the procedure.

All patients were followed up at our outpatient clinic every month for 3 months after the procedure, and thereafter every 2‐3 months for 9 months after the procedure. Oral anticoagulants were maintained for at least 3 months after the procedure. AADs except beta‐blockers were continued for 1‐2 months and then discontinued if the patient had no recurrence of AF/AT. The recurrence of AF/AT was evaluated based on clinical symptoms and ECG, including 12‐lead ECG at every visit, 24‐hour Holter ECG at 3 and 12 months, and 30‐s ECG recorded with a mobile event recorder at a minimum of 1‐2 times a day for 3‐6 months after the procedure. Recurrence of AF/AT defined as any episode of atrial tachyarrhythmia lasting > 30 s after 3 months of the blanking period without AAD.

### 2.3 Statistical analysis

Continuous data are given as the mean ± SD. All reported *P*‐values are two‐sided, and *P* < .05 was taken to indicate statistical significance. Statistical analyses were performed using spss 19.0 (SPSS Inc).

First, total patients were divided into those who underwent CA (Ablation group) and who did not (No‐ablation group). The differences in continuous and categorical variables between the two groups were tested by unpaired Student's t test and χ^2^ test, respectively. To identify the clinical variables that affected CA performance, a multivariate model of the logistic regression analysis was developed. In this model, clinical factors that are listed as the major issues to affect the indication of CA in recent guidelines^4^, ^5^, ^6^, ^7^ (age ≥ 75 years, NYHA class ≥ II, long‐standing persistent AF, asymptomatic AF, female sex, left atrial diameter ≥ 50 mm, left ventricular ejection fraction < 40%, and cardiomyopathy) were forcedly introduced. Assigning 1 point for each independent variable, we developed a new scoring system. The predictive capability of the new score for the incidence of CA performance was evaluated by the area under the receiver operating characteristic (ROC) curve.

Second, the effects of the new score on the procedural results of CA and the rhythm outcome after initial CA were evaluated. The cumulative event‐free rate of recurrent AF/AT was estimated by the Kaplan–Meier method, and the difference by the score was tested by the log‐rank test.

Third, to understand the precise mechanisms in the relationship between the new score and the procedural results or the rhythm outcome, further analyses were performed using the components of the scoring system. The effect of the components on non‐PV triggers and substrate modification during CA were evaluated by the multivariable models with logistic regression analysis. The effect of the components on AF/AT recurrence was evaluated by the univariate and multivariate models with Cox regression analysis.

## 3 RESULTS

### 3.1 Patient characteristics

Among the 2898 AF patients, CA was performed in 938 (32.4%) after the initial visit. Table 1 shows the patient characteristics in overall patients, patients who underwent CA (Ablation group), and patients who did not undergo CA (No‐ablation group). Patients in the no‐ablation group were older (*P* = .001) and more female (*P* < .001), and had lower body mass index (*P *< .001), higher B‐type natriuretic peptide (BNP) (*P *< .001), lower estimated glomerular filtration (eGFR) (*P *= .003) and larger left atrial diameter (LAD) (*P *< .001) compared with those in the ablation group. Moreover patients in the no‐ablation group had a higher prevalence of long‐standing persistent AF (*P* < .001), asymptomatic AF (*P* < .001), NYHA class ≥ II (*P* < .001), and organic heart diseases (*P* < .001), and inversely had a less prevalence of paroxysmal AF (*P *= .001) and a less usage of class I AAD (*P *< .001) and class III AAD compared with those in the ablation group.

**TABLE 1:** Patient characteristics

	Overall	Ablation	No‐ablation	*P*‐value
Number of patients	2898	938	1960	NA
Age (years)	63.6 ± 12.7	58.7 ± 10.7	65.9 ± 13.0	<0.001
Age ≥ 75 years	582 (20.1)	36 (3.8)	546 (27.9)	<0.001
Female sex	718 (24.8)	131 (17.2)	557 (28.4)	<0.001
Hypertension	1389 (47.9)	412 (43.9)	977 (49.8)	0.003
Diabetes mellitus	438 (15.1)	93 (9.9)	345 (17.6)	<0.001
Organic heart diseases	746 (25.7)	103 (11.0)	643 (32.8)	<0.001
Ischemic heart disease	203 (7.0)	24 (2.6)	179 (9.1)	<0.001
Valvular heart disease	457 (15.8)	40 (4.3)	417 (21.3)	<0.001
Cardiomyopathy	169 (6.5)	41 (4.4)	148 (7.6)	0.001
NYHA class ≥ II	386 (13.3)	23 (2.5)	363 (18.5)	<0.001
Previous cerebral infarction/TIA	157 (5.4)	39 (4.2)	118 (6.0)	0.038
CHADS2 score	1.08 ± 1.12	0.67 ± 0.78	1.27 ± 1.20	<0.001
CHA2DS2‐VASc score	1.87 ± 1.59	1.18 ± 1.13	2.20 ± 1.68	<0.001
Paroxysmal AF	1767 (61.0)	613 (65.4)	1154 (58.9)	0.001
Persistent AF	608 (21.0)	254 (27.1)	354 (18.1)	<0.001
Long‐standing persistent AF	525 (18.0)	69 (7.4)	456 (23.3)	<0.001
Asymptomatic AF	1085 (37.4)	243 (25.9)	842 (43.0)	<0.001
Body mass index	20.5 ± 9.2	21.8 ± 8.0	19.8 ± 9.7	<0.001
BNP (pg/mL)	204 ± 416	102 ± 131	257 ± 495	<0.001
eGFR (mL/min/1.73 m^2^)	65.7 ± 17.2	69.3 ± 14.1	63.7 ± 18.5	<0.001
Left ventricular ejection fraction (%)	62.5 ± 11.8	63.6 ± 9.8	61.9 ± 12.6	<0.001
Left atrial diameter (mm)	41.2 ± 8.3	39.6 ± 6.5	41.9 ± 9.0	<0.001
Left atrial diameter ≥ 50mm	360 (12.4)	55 (5.9)	305 (15.6)	<0.001
Class I antiarrhythmic drug	887 (30.6)	498 (53.1)	389 (19.8)	<0.001
Class III antiarrhythmic drug	152 (5.2)	69 (7.4)	83 (4.2)	<0.001

Values are shown as mean ± SD or n (%).

Abbreviations: AF, atrial fibrillation; BNP, B‐type natriuretic peptide; CHA2DS2‐VASc score, congestive heart failure = 1, hypertension = 1, age ≥ 75 years = 2, diabetes = 1, stroke/TIA = 2, vascular disease = 1, age 65‐74 years = 1, and female sex = 1; CHADS2 score, congestive heart failure = 1, hypertension = 1, age ≥ 75 years = 1, diabetes = 1, and stroke/TIA = 2; eGFR, estimated glomerular filtration; NA, not applicable; NYHA, New York Heart Association; TIA, transient ischemic attack.

### 3.2 Development of HEAL‐AF score

Table 2 shows the results of the logistic regression analysis which determined the predictors for CA. In the multivariate model, age ≥ 75 years (OR 0.142, 95% CI 0.098‐0.204, *P *< .001), HF ≥ NYHA II (OR 0.173, 05% CI 0.109‐0.272, *P* < .001), asymptomatic AF (OR 0.529, 95% CI 0.435‐0.643, *P *< .001), long‐standing persistent AF (OR 0.396, 95% CI 0.294‐0.533, *P* < .001), female sex (OR 0.634, 95% CI 0.506‐0.795, *P* < .001), and LAD ≥ 50 mm (OR 0.699, 95% CI 0.469‐0.984, *P *= .040) were independent predictors for CA.

**TABLE 2:** Multivariate logistic regression model for predicting the selection of catheter ablation performance

	OR	95% CI	*P*‐value
Age ≥ 75 y	0.142	0.098‐0.204	<0.001
NYHA class ≥ II	0.173	0.109‐0.272	<0.001
Long‐standing persistent AF	0.396	0.294‐0.533	<0.001
Asymptomatic AF	0.529	0.435‐0.643	<0.001
Female sex	0.634	0.506‐0.795	<0.001
Left atrial diameter ≥ 50 mm	0.699	0.469‐0.984	0.04
Left ventricular ejection fraction < 40%	0.825	0.508‐1.340	0.438
Cardiomyopathy	1.243	0.809‐1.911	0.321

Abbreviations: AF, atrial fibrillation; CI, confidence interval; OR, odds ratio.

With the acronym of the six independent variables of **H**eart failure (NYHA class ≥ II), **E**lderly patients (age ≥ 75 years), **A**symptomatic AF, **L**ong‐standing persistent AF, left **A**trial dilation (LAD ≥ 50 mm), **F**emale sex, we developed a new HEAL‐AF scoring system. For easy to use, the HEAL‐AF score is the sum of the points assigned 1 for each of the 6 variables (ranged 0‐6 points).

**Figure 1:**
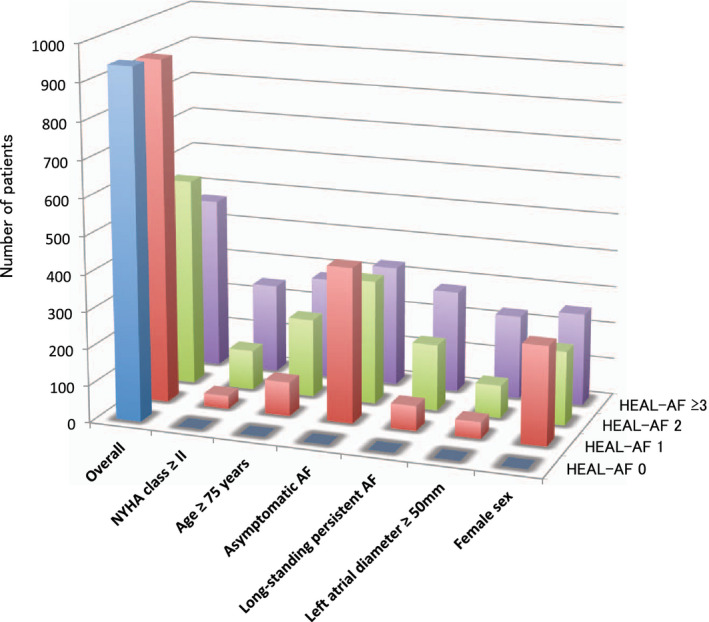
Distribution of patients according to the number of HEAL‐AF score and each components of the HEAL‐AF score. HEAL‐AF score, NYHA class ≥ II = 1, age ≥ 75 years = 1, asymptomatic AF = 1, long‐standing persistent AF = 1, left atrial diameter ≥ 50 mm = 1, female sex = 1

**Figure 2:**
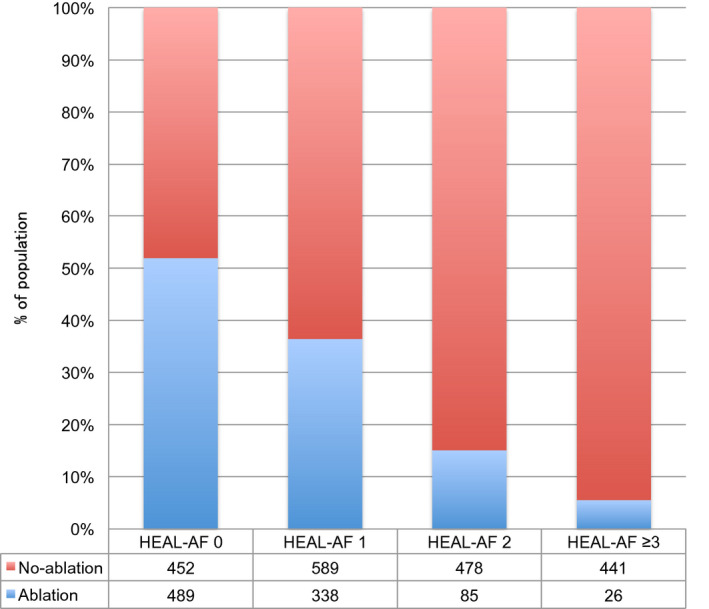
Prevalence of patients undergoing catheter ablation according to the HEAL‐AF score. HEAL‐AF score, NYHA class ≥ II = 1, age ≥ 75 years = 1, asymptomatic AF = 1, long‐standing persistent AF = 1, left atrial diameter ≥ 50 mm =1, female sex = 1; Ablation, patients who underwent catheter ablation; No‐ablation, patients who did not underwent catheter ablation

For further analysis, patients were classified into following four groups according to the HEAL‐AF score: 0 point (HEAL‐AF 0), 1 point (HEAL‐AF 1), 2 points (HEAL‐AF 2), and 3 or more points (HEAL‐AF ≥ 3). Figure 1 shows the distribution of patients according to the classification of HEAL‐AF and each component of the HEAL‐AF score. Among the 2898 patients, 941 (32.5%), 927 (32.0%), 563 (19.4%), and 467 (16.1%) were classified as HEAL‐AF 0, 1, 2, ≥3, respectively. The distribution of patients in the ablation and in the non‐ablation group according to the HEAL‐AF score is shown in Figure 2. The prevalence of the patients in the ablation group was 52.0% in HEAL‐AF 0, 36.5% in HEAL‐AF 1, 15.1% in HEAL‐AF 2, and 5.6% in HEAL‐AF ≥ 3. The predictive capabilities of the HEAL‐AF score for CA as determined by the area under the curve (AUC) was 0.720 (95% CI 0.701‐0.739, *P* < .001).

### 3.3 **HEAL‐AF score and results of** CA

**Figure 3:**
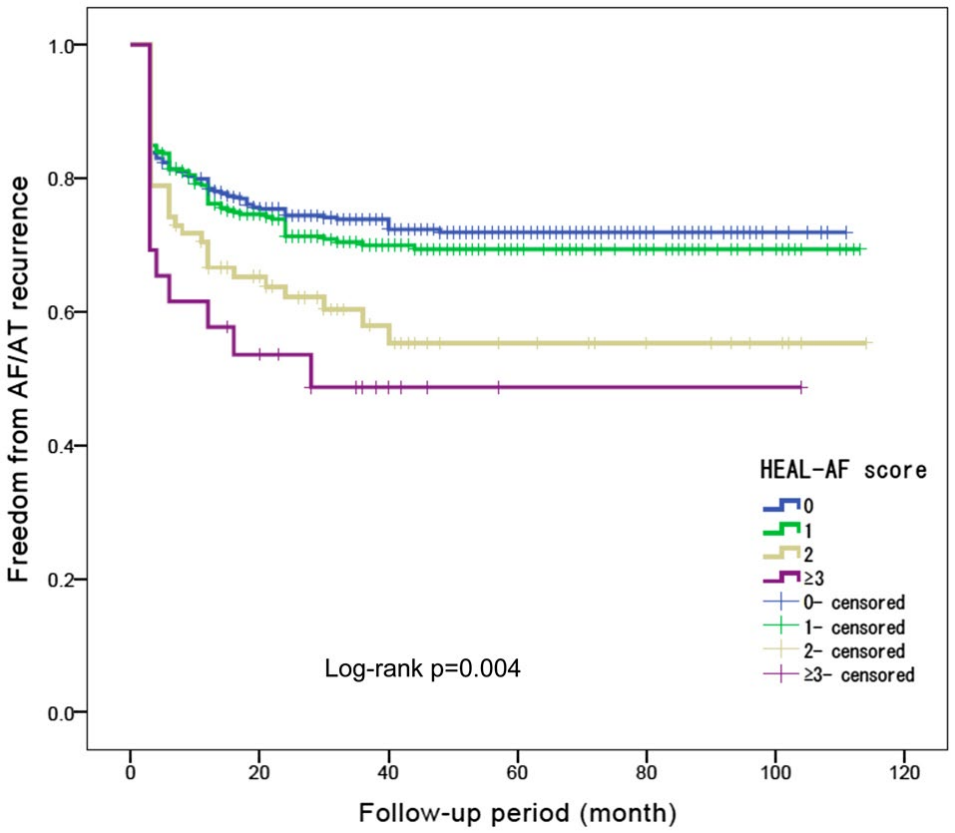
Kaplan–Meier estimate curve of freedom from AF/AT recurrence after a single procedure. HEAL‐AF score, NYHA class ≥ II = 1, age ≥ 75 years = 1, asymptomatic AF = 1, long‐standing persistent AF = 1, left atrial diameter ≥ 50 mm = 1, female sex = 1. AF, atrial fibrillation; AT, atrial tachycardia

Table 3 shows the procedural results of CA according to the HEAL‐AF score in patients of the ablation group (n = 938). As HEAL‐AF score increased, total procedure time, fluoroscopic time, and ablation time became longer (all, *P* < .001), and the frequency of substrate modification (*P* < .001), including CFAE ablation (*P* = .007) and linear ablation in LA (*P* < .001), significantly increased. Figure 3 shows the Kaplan‐Meier curves of the event‐free rate of AF/AT recurrence according to the HEAL‐AF score (mean follow‐up period, 36.5 ± 28.8 month). The cumulative event‐free rates of AF/AT recurrence after initial CA at the end of the follow‐up were 73.2% in HEAL‐AF 0, 71.0% in HEAL‐AF 1, 60.0% in HEAL‐AF 2, and 50.0% in HEAL‐AF ≥ 3 (log‐rank test, *P* = .004). The hazard ratios (HRs) of the HEAL‐AF score for AF/AT recurrence (non‐adjusted) with HEAL‐AF 0 as a reference are shown in Table 4. HEAL‐AF 1 (HR 1.141, 95% CI 0.868‐1.498, *P* = .344) was not significantly associated with AF/AT recurrence, whereas HEAL‐AF 2 (HR 1.755, 95% CI 1.192‐2.583, *P* = .004) and ≥ 3 (HR 2.211, 95% CI 1.246‐3.923, *P* = .007) were significantly associated with AF/AT recurrence.

**TABLE 3:** Procedural results according to HEAL‐AF score in patients with ablation group (n = 938)

HEAL‐AF	0	1	2	≥3	*P*‐value
Number of patients	489	338	85	26	NA
Total procedure time (min)	145 ± 41	150 ± 40	167 ± 48	168 ± 41	<0.001
Fluoroscopic time (min)	27 ± 10	28 ± 10	32 ± 11	35 ± 12	<0.001
Ablation time (min)	46 ± 19	49 ± 21	58 ± 24	55 ± 18	<0.001
Additional ablation					
Non‐PV triggers	89 (18.2)	55 (16.3)	14 (16.5)	7 (26.2)	0.539
Substrate modification	135 (29.7)	141 (44.3)	48 (60.0)	13 (50.0)	<0.001
CFAE ablation	98 (20.0)	87 (25.7)	31 (36.5)	6 (23.1)	0.007
Linear ablation	77 (15.7)	92 (27.2)	38 (44.7)	10 (38.5)	<0.001

Values are shown as mean ± SD or n (%). HEAL‐AF score, NYHA class ≥ II = 1, age ≥ 75 years = 1, asymptomatic AF = 1, long‐standing persistent AF = 1, left atrial diameter ≥ 50 mm = 1, female sex = 1.

Abbreviations: CFAE, complex fractioned atrial electrogram; NA, not applicable; PV, pulmonary vein.

**TABLE 4:** Cox regression models for predicting AF/AT recurrence

	Univariate			Multivariate		
	HR	95% CI	*P*‐value	HR	95% CI	*P*‐value
HEAL‐AF 1	1.141	0.868‐1.498	0.344			
HEAL‐AF 2	1.755	1.192‐2.583	0.004			
HEAL‐AF ≥ 3	2.211	1.246‐3.923	0.007			
Compornents of HEAL‐AF						
Age ≥ 75 years	0.85	0.437‐1.625	0.632			
NYHA class ≥ II	0.601	0.224‐1.614	0.313			
Long‐standing persistent AF	1.869	1.299‐2.689	0.001	1.858	1.291‐2.674	0.001
Asymptomatic AF	1.241	0.958‐1.609	0.102			
Female sex	1.378	1.035‐1.835	0.028	1.37	1.029‐1.824	0.031
Left atrial diameter ≥ 50mm	1.184	0.733‐1.911	0.49			
Other possible variables						
Diabetes mellitus	0.887	0.588‐1.338	0.569			
Left ventricular ejection fraction < 40%	0.875	0.413‐1.854	0.727			
Cardiomyopathy	0.929	0.508‐1.697	0.81			
Ischemic heart disease	1.591	0.846‐2.993	0.15			
eGFR < 60 mL/min/1.73 m^2^	1.068	0.809‐1.411	0.642			
Body mass index ≥ 25	0.799	0.615‐1.037	0.092			

Abbreviations: AF, atrial fibrillation; AT, atrial tachycardia; CI, confidence interval; eGFR, estimated glomerular filtration; HEAL‐AF score, NYHA class ≥ II = 1, age ≥ 75 years = 1, asymptomatic AF = 1, long‐standing persistent AF = 1, left atrial diameter ≥ 50 mm = 1, female sex = 1; HR, Hazard ratio.

### 3.4 **Components of HEAL‐AF score and results of** CA

Table 5 shows the multivariate logistic regression models for predicting non‐PV triggers and substrate modification using the components of the HEAL‐AF score in patients of the ablation group (n = 938). Female sex (OR 1.706, 95% CI 1.153‐2.686, *P* = .009) was the independent predictor of non‐PV triggers, and asymptomatic AF (OR 2.144, 95% CI 1.568‐2.931, *P* < .001), long‐standing persistent AF (OR 2.150, 95% CI 1.265‐3.654, *P* = .005, and LAD ≥ 50 mm (OR 1.061, 95% CI 1.081‐3.534, *P* < .025) were independent predictors of substrate modification. Cox regression models for AF/AT recurrence using the components of HEAL‐AF score is shown in Table 4. Long‐standing persistent AF (HR 1.858, 95% CI 1.291‐2.674, *P* = .001) and female (HR 1.370, 95% CI 1.029‐1.824, *P* = .031) were independent predictors of AF/AT recurrence in the multivariate analysis.

**TABLE 5:** Multivariate logistic regression models for predicting non‐PV triggers and substrate modification

	Non‐PV triggers			Substrate modification		
	OR	95% CI	*P*‐value	OR	95% CI	*P*‐value
NYHA class ≥II	0.584	0.163‐2.097	.410	1.110	0.455‐2.708	.818
Age ≥75 years	1.014	0.429‐2.395	.975	1.650	0.825‐3.299	.157
Asymptomatic AF	0.636	0.411‐0.984	.042	2.144	1.568‐2.931	<.001
Long‐standing persistent AF	0.960	0.482‐1.911	.906	2.150	1.265‐3.654	.005
Left atrial diameter ≥ 50mm	1.558	0.755‐3.135	.214	1.961	1.088‐3.534	.025
Female sex	1.760	1.153‐2.686	.009	0.966	0.664‐1.405	.856

Abbreviations: AF, atrial fibrillation; CI, confidence interval; OR, odds ratio; PV, pulmonary vein.

### 3.5 Other clinical variables for predicting AF/AT recurrence

Table 4 also shows Cox regression models for predicting AF/AT recurrence using the other possible clinical variables, including diabetes mellitus, left ventricular ejection fraction < 40%, cardiomyopathy, ischemic heart disease, eGFR < 60 mL/min/1.73 m^2^, and body mass index ≥ 25. Among these variables, there were no significant predictors in the univariate analysis.

### 3.6 **Adverse outcomes after** CA

Pericardial effusion requiring pericardiocentesis after the ablation procedure occurred in two patients with HEAL‐AF 0 and 1 patient with HEAL‐AF 1 (asymptomatic AF). Symptomatic stroke after the ablation procedure occurred in 1 patient with HEAL‐AF 1 (female). There were no patients complicated by symptomatic PV stenosis or atrioesophageal fistula.

## 4 DISCUSSION

### 4.1 Main findings

In this single hospital‐based cohort study, we identified the independent predictors for the actual CA performance in patients with AF, consequently developing a new scoring system. The HEAL‐AF score, which are based on simple and easily obtained clinical variables, could predict the incidence of CA with a moderate predictive value. In addition, the HEAL‐AF score was associated with complex ablation strategy, and AF/AT recurrence after CA.

### 4.2 Clinical implications of HEAL‐AF score

First, the HEAL‐AF score was significantly associated with the CA incidence of AF in the present study. Although recent guidelines^4^, ^5^, ^6^, ^7^ have demonstrated that types and duration of AF, degree of symptoms, concomitance of significant HF, and older age are major issues for CA indication, it has not been well established how to select appropriate patients for CA in AF population. For the HEAL‐AF scoring system, we adopted 6 variables, including these 4 issues, which independently associated with the incidence of CA performance, thereby stratifying the physicians' selection tendency with CA in AF patients. In addition, patient preference for CA is one of the most important factors in the decision regarding CA, especially in patients with asymptomatic AF and elderly patients, which was incorporated into the new score. Therefore, the HEAL‐AF score could reflect both medical judgment and possibly also patient preference in CA.

Second, the present study demonstrated a significant association between the HEAL‐AF score and the outcome of CA. As HEAL‐AF score increased, the frequency of substrate modification was significantly increased in the present study. Moreover, the HEAL‐AF score was significantly associated with AT/AT recurrence after the CA procedure: compared with HEAL‐AF‐0, HEAL‐AF 1 had a similar incidence, while HEAL‐AF 2 and ≥ 3 had 1.8 and 2.2 fold incidence of AF/AT recurrence, respectively. Interestingly, some components of the HEAL‐AF score overlapped with other scoring systems predicting low voltage zone in LA,^13^ and AF recurrence after CA.^14^, ^15^

Therefore, the HEAL‐AF score classification could provide a measure to select not only suitable patients, but also favorable outcomes with suitable strategies for CA of AF. In addition, due to the unique features of simple and easily obtained clinical variables, without multinominal categories of age and left atrial diameter, and without assessing renal function, the HEAL‐AF score may gain an advantage as a more convenient tool in the clinical practice over other scoring systems.^13^, ^14^, ^15^ In patients with HEAL‐AF 0, CA, especially the PVI only strategy, will be adequate in most patients. In patients with HEAL‐AF 1, CA may be reasonable with a sufficient procedural success rate, but additional ablation strategy beyond PVI may considered in some patients. In patients with HEAL‐AF 2, careful patient judgment and specific ablation strategies will be required. In patients with HEAL‐AF ≥ 3, CA for AF was less experienced in the present study. Indication of CA in this population should be more carefully considered because of the possibly limited efficacy.

### 4.3 HEAL‐AF score and performance of CA in HF patients

Several randomized trials in patients with AF and HF have reported improvement in both soft endpoints (improving LVEF and maintaining sinus rhythm) and hard endpoints (death and hospitalization for heart failure) by CA.^16^, ^17^, ^18^ Most patients enrolled in these trials have generally been younger men with paroxysmal or persistent AF, who would be classified as HEAL‐AF 1 in the present study. These patients would belong to HEAL‐AF 1 who have HF without other components of the HEAL‐AF score, which suggest favorable outcome. There are controversies regarding the procedural strategy of CA in patients with HF. The AATAC trial,^17^ mostly including patients with possible HEAL‐AF 1, demonstrated that patients undergoing PVI and posterior wall isolation had significantly better outcome than patients with PVI alone. In contrast, the CAMTAF trial,^16^ mostly including patients with possible HEAL‐AF 2 or more, showed that single procedure success rate at 1 year was 38% when additional CFAE ablation and/or linear ablation over PVI were performed. These results are almost consistent with the present results. However, as shown in Table 1, number of HF patients classified as HEAL‐AF 1 was relatively small. In other words, most of HF patients were classified as HEAL‐AF 2 or more. Consequently, CA performance rate in HF patients might be low in the present study. In addition, HF patients were comprehensively analyzed regardless of left ventricular function and valvular heart disease, because of the adoption of NYHA class ≥ II in the HEAL‐AF score. Further studies are necessary to evaluate the optimal indication and strategy of CA in AF patients with HF, especially when the patients have other HEAL‐AF components.

### 4.4 HEAL‐AF score and performing CA in elderly and female patients

Although there was no significant association between AF/AT recurrence and elderly patients in the present study, the efficacy of CA for elderly patients has remained controversial. A pooled analysis reported by Kautzner et al^19^ showed that the success rate of a single CA procedure was not different between older and younger groups. On the other hand, in several scoring system to predict AF recurrence after CA, older age has been introduced as one of the most common predictors.^13^, ^14^, ^15^ Metzner et al^20^ reported that CA in elderly patients is associated with a favorable long‐term outcome in patients with paroxysmal AF, while results are less promising in patients with persistent or long‐standing persistent AF, classified as HEAL‐AF 2 or more, irrespective of multiple CA procedure. In the present study, most of elderly patients had 2 or more components of HEAL‐AF score. Consequently, CA performance for elderly patients might be limited. In addition, other clinical variables, including frailty and comorbidities (such as Charlson comorbidity index^21^), may also affect the decision making for CA in elderly patients. To determine the optimal indication of CA for elderly patients, more specific approach will be needed.

The present study also showed that female sex was an independent predictor of AF/AT recurrence. Interestingly, several studies showed an increased rate of AF recurrence in women after CA.^22^, ^23^, ^24^ Reasons for this gender difference may be explained which are categorized into patient selection bias and AF mechanisms. Several studies showed that women who underwent CA are older, have high comorbidities, have higher rate of long‐standing persistent AF, and referred for CA later in their clinical course.^25^, ^26^ In addition, similar to the present study, Patel et al^26^ reported that women had more non‐PV triggers than men.

### 4.5 HEAL‐AF score and performing CA in asymptomatic AF or long‐standing persistent AF patients

The prevalence of asymptomatic AF has been reported to be 10%‐40% in a variety of cohorts.^27^ For this large population of asymptomatic AF, the indication of CA is still controversial. Forleo et al^28^ found similar success rates of ablation between symptomatic (mostly classified as HEAL‐AF 0) and asymptomatic patients (mostly classified as HEAL‐AF 1) with paroxysmal and persistent AF. On the other hand, in a study by Wu et al^29^ the success rates of ablation were much lower in asymptomatic patients with persistent or long‐standing persistent AF (mostly classified as HEAL‐AF 2) than in a matched group of symptomatic patients (mostly classified as HEAL‐AF 1). In the present study, asymptomatic AF was not significantly associated with AT/AT recurrence, while long‐standing persistent AF was an independent predictor of AF/AT recurrence. Therefore, for younger asymptomatic AF patients, classified as HEAL‐AF 1 in the present study, CA could be considered appropriate before or soon after progression to long‐standing persistent AF. Asymptomatic AF, as well as long‐standing persistent AF and LAD ≥ 50 mm were independent predictors of substrate modification in the present study. Further studies are necessary to determine the optimal CA strategy for patients who have these HEAL‐AF components.

### 4.6 Study Limitations

The present study had several limitations. First, our database consisted of patients from a single cardiovascular hospital which included only Japanese patients. In addition, patients in our cohort were younger than those in other areas or population‐based registries.^30^, ^31^ Therefore, the results should be interpreted carefully when applied to different populations. Second, although CFAE ablation and left atrial linear ablation were performed as substrate modification, the present study could not address which is better strategy for substrate modification. Third, findings of repeat ablation procedures in patients with AT/AF recurrence could not be shown in the present study, because repeat ablation procedures were not fully performed in all patients with recurrent AF/AT. Fourth, although all patients underwent daily 30‐s ECG recording with a mobile event recorder and Holter recordings at prescribed intervals, asymptomatic episodes of AF/AT may sometimes have been missed, and less rigorous follow‐up methods may have overestimated the results.

## 5 CONCLUSIONS

The HEAL‐AF score, which is based on simple and easily obtained clinical variables, was significantly associated with CA for and AF/AT recurrence after CA in patients with AF. In patients with HEAL‐AF 0, CA, with the PVI only strategy, will be useful for most patients; In HEAL‐AF 1, CA may be reasonable with sufficient procedural success rate; In HEAL‐AF 2, careful patient judgment and specific ablation strategy should be considered; In HEAL‐AF ≥ 3, CA for AF was less experienced.

## DISCLOSURES

The Institutional Review Board of the Cardiovascular Institute approved the study (Date of IRB approval; May, 2019; Approval number, 169).

## CONFLICT OF INTEREST

The following authors have potential conflicts of interest: SS received research funding from Daiichi‐Sankyo and Mitsubishi‐Tanabe. TI received research funding from Medtronic Japan, Japan Lifeline, and Daiichi‐Sankyo, and remuneration from Ono Pharmaceutical, Bayer, Daiichi‐Sankyo, Nippon Boehringer Ingelheim, and Toa Eiyo. TY received research funding from Daiichi‐Sankyo, Bayer, and Bristol Myers Squibb, and remuneration from Daiichi‐Sankyo, Bayer, Bristol Myers Squibb, Pfizer, Nippon Boehringer Ingelheim, Ono Pharmaceutical, and Toa Eiyo.
